# Transcranial Direct Current Stimulation Decreases the Decline of Speed during Repeated Sprinting in Basketball Athletes

**DOI:** 10.3390/ijerph18136967

**Published:** 2021-06-29

**Authors:** Che-Hsiu Chen, Yu-Chun Chen, Ren-Shiang Jiang, Lok-Yin Lo, I-Lin Wang, Chih-Hui Chiu

**Affiliations:** 1 Department of Sport Performance, National Taiwan University of Sport, Taichung 404, Taiwan; jakic1114@ntupes.edu.tw; 2 Department of Physical Education, National Taiwan University of Sport, Taichung 404, Taiwan; ycchen1016@ntupes.edu.tw (Y.-C.C.); jiangrs@ntus.edu.tw (R.-S.J.); 3 Graduate Program in Department of Exercise Health Science, National Taiwan University of Sport, Taichung 404, Taiwan; 10706002@gm.ntupes.edu.tw; 4 College of Physical Education, Hubei Normal University, Huangshi 435002, China; ilin@gms.ndhu.edu.tw

**Keywords:** fatigue, ATP-PC, power, countermovement jump, sprint

## Abstract

The purpose of this study was to determine whether transcranial direct current stimulation (tDCS) can improve countermovement jump performance, fatigue index and alleviate the speed decline during repeated shuttle sprints in trained basketball players. Thirteen trained basketball players were divided into the tDCS trial and sham trial by the random crossover design. The tDCS trial was stimulated with 2-mA current in the M1 area in the middle of the top of the head for 20 min. For the sham trial, the current was turned off after 5 s, stopping the electrical stimulation. After warming up, the players underwent countermovement jump test, weighted countermovement jump test and then performed 40 × 15-m sprints with with a 1:4 exercise: rest ratio. The jump height, sprinting time, fatigue index, heart rate and rating of perceived exertion (RPE) were analyzed by paired-sample t-test, when significance was discovered by two-way repeated measures analysis of variance. The study results revealed that the tDCS trial significantly increase the countermovement jump performance (*p* = 0.04), decrease the sprinting time (*p* = 0.016), and had improved fatigue index during the sprinting process (*p* = 0.009). However, the heart rate and RPE during sprinting were nonsignificantly different between the trials. This study has identified that tDCS can decrease the speed decline, fatigue index during sprinting and increase countermovement jump performance without affecting heart rate or the rating of perceived exertion.

## 1. Introduction

In transcranial direct current stimulation (tDCS), which is a noninvasive brain stimulation, a weak and continuous electric current is employed to stimulate the scalp and is transmitted to the brain [1]. Previously, the method was mainly used to treat medical conditions, such as depression and Parkinson’s disease, and in rehabilitation after stroke [2,3,4]. To date, some researchers have demonstrated that tDCS has positive effects on limb movement and function [5,6]. Consequently, several studies have indicated that tDCS can increase the excitability of the brain’s motor cortex, and thus, improve exercise performance [7,8].

Some studies reported that the use of 1–2 mA tDCS for 10 to 20 min before exercise activated the motor cortex and sequentially improved the isometric contraction ability, muscle strength, and power of the participants [6]. In another study, a 20-min session of tDCS maintained favorable exercise performance in three consecutive days of high-intensity resistance training in untrained participants. These participants also had lower perceived fatigue during exercise when they had undergone tDCS. Whereas, they had higher perceived fatigue when resting after exercise [9]. Therefore, tDCS can improve performance in resistance exercise. In addition, in people with considerable weight-training experience, a 20-min tDCS session can improve countermovement jump height and power [5,6].

In repeated intermittent sprinting, improving power and speed endurance, including increasing the vertical jump height and reducing muscle fatigue caused by sprinting are crucial to improving exercise performance. Previous studies have found that tDCS increases muscle endurance in a single-joint isometric exercise [10,11]. In a study of intermittent exercise, participants received tDCS for 20 min and then did five 6-s cycling sprints at maximum effort and 24-s breaks between sprints. The tDCS was found to significantly improve participants’ sprinting performance and cognitive functions [8]. In the study of Lee et al., the power, agility, and balance of participants was tested after they received tDCS, and tDCS was found to significantly improve the participants’ agility and exercise ability related to the lower limbs [12]. A 20-min tDCS session was shown to improve the countermovement jump height and power of individuals with advanced weight-training experience [6], as well as their muscle activation level, agility, and sprinting performance. Thus, tDCS has significant positive effects on the performance of muscle-strength- and power-oriented exercise.

Phosphagen system capacity is extremely crucial for the performance of high-intensity interval exercise. Such capacity of trained athletes can be effectively measured by completing 40 15-m sprints at a sprint:break ratio of 1:4 [13]. In basketball, training and games require that players perform numerous repeated shuttle sprints. A high sprinting speed is crucial to the performances of basketball players. Only a few studies have investigated the influence of tDCS on high-intensity interval exercise involving cycling. tDCS was discovered to alleviate the exercise performance decline caused by fatigue [8]. However, whether it influences performance in repeated sprints is yet to be determined. Additionally, no research has identified whether tDCS improves countermovement jump ability and high-intensity interval exercise performance in trained basketball players. The goal of this study was to determine whether tDCS can improve countermovement jump performance, fatigue index, reduce the decline in speed, heart rate and RPE of trained basketball players during repeated shuttle sprints.

## 2. Materials and Methods

### 2.1. Participants

Thirteen healthy male adult basketball players who trained regularly were recruited (age: 20.3 ± 0.64 years; height: 180.6 ± 7.3 cm; weight: 73.2 ± 7.4 kg; body fat: 13.5% ± 2.7%). During recruitment, the authors inquired about their physical condition and current or past injuries. Individuals who had major lower-limb injuries and who had lower-limb injuries at the time of the study recruitment were excluded. Prior to the start of the study, participants were informed about the experiment process and possible problems that they may encounter. In addition, a written informed consent document was obtained from all participants. This study was approved by the Institutional Review Board of Jilin Sport University (JLSU-IRB2020001).

### 2.2. Experiment Design

The single-blind (participants were blinded) randomized crossover design was adopted for this study. The participants were divided into the tDCS and sham trial groups. Once the first experiment had been completed, the participants were instructed to rest for 7–10 days before the next experiment. They were further required to record all training schedule and repeat the same training schedule 7–10 days before the next experiment. All tests were expected to be completed within 1 month. During the experiment period, all the participants maintained their regular training routine with no changes to their basketball and weight training. In addition, they did not engage in excessive training or additional games. After two simulation tests, the first round of the actual tests was conducted.

### 2.3. Experiment Procedure

Prior to the actual experiment, each participant received at least two sport-skill-specific tests and a simulation test of 15-m sprints to familiarize themselves with the process of the actual tests. The participants had to record their diet on the three days before the first round of the actual tests. In addition, they had to have a similar diet on the three days preceding the following rounds of tests. On the day of the experiment, all participants were provided with the same breakfast to ensure that they had the same energy intake.

All the experiments were begun at 8 am. After the participants had arrived at the laboratory, they were given a fixed breakfast and then instructed to rest for 1 h. At approximately 9 am to 9:30 am, the participants put on the Halo Sport (Halo Neuroscience, San Francisco, CA, USA). The current intensity was adjusted to 2 mA, and the current was conducted for 20 min in the tDCS group. The Halo Sport is a machine with a shape similar to that of headphones. It has three 24 cm^2^ primers to connect the user to the current. There are two different type of stimulation with tDCS. Anodal stimulation mainly acts to excite neuronal activity, while cathodal stimulation reduces or inhibits neuronal activity. In the present study, the anodal electrode was only used and placed on CZ, C5, and C6 according to the 10–20 EEG system. The tDCS provided stimulate both the hemispheres of the motor cortex. In the sham group, the current was stopped manually with iPad once it had been on for 5 s, stopping the electrical stimulation. This is the same stimulation procedure, as used in previous studies of tDCS [8,14].

During the process of electrical stimulation or sham stimulation, upbeat music was played through Bluetooth headphones to divert the participants’ attention.

After the electrical stimulation or sham simulation, the participants put on a heart rate watch (polar, Oy, Finland) and rested for 10 min, after which their resting heart rate was measured. The participants subsequently began to perform dynamic stretches and warm-up. Each set of dynamic stretches comprised six stretches, and the set order was the quadricep stretch, posterior thigh muscle stretch, gluteal muscle stretch, lunge with the arms raised overhead and body leaning backward, lunge with twist, side lunge, and shoulder rotation. After dynamic stretching, they performed six—for the left and right legs each alternately—skip steps, high knees, forward skips, side steps, stride jumps, and single-leg side jumps. Then, they performed a 15-m acceleration sprint for four times. Finally, they finished the warm up by doing 10 squat jumps.

**Figure 1:**
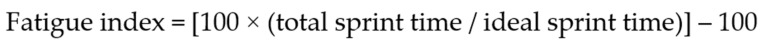
The formula of fatigue index. Total sprint time = sum of sprint times from all sprint. Ideal sprint time = the number of sprints × fastest sprint time.

After the warm-up, the power test, in which the participants performed countermovement jumps, was conducted. Each test involved three jumps with a break of at least 30 s between each jump. The average height of the three jumps was used as the power index. In the weighted countermovement jump, the participants held a 10 kg dumbbell in each hand, with the total weight being 20 kg. When they were ready, they squatted and jumped up with full strength. The change in CMJ height/weighted CMJ height (%) was employed to determine the maximum muscle strength of the participants [15]. Gymaware (Gymaware, KineticPerformance, Australia) was used for measuring the jump height; the device was fixed on a belt around the participant’s waist. After the power test, participants performed 40 × 15-m sprints at the exercise:rest ratio of 1:4 for the high-intensity interval exercise test. After a participant sprint, the timer records the result and calculated the rest time. For example, if the exercise time is 2 s, the rest time is 8 s. This test method was previously found to be able to determine phosphagen system capacity [6]. During the test process, the timing gate system (Witty, Microgate, Italy) was used to document the sprint time, and the break time was immediately calculated. A 5-s countdown was begun 5 s before the break time ended to ensure that the sprint:break ratios of the participants were identical. The participants’ heart rate before and after each sprint and their rating of perceived exertion (RPE) values before the sprint (0) and after the 10th, 20th, 30th, and 40th sprints were documented. Fatigue index (Figure 1) was calculated using the formula of Hughes (2006) [16]:

### 2.4. Statistical Analysis

The data in this study are displayed as the mean ± standard deviation. The Shapiro–The Wilk test was first conducted to examine the normality of the data. The countermovement jump height, sprint time, RPE, and heart rate of the two groups were analyzed using two-way repeated measures analysis of variance. When significance was discovered, the difference between tDCS group and sham group on the countermovement jump heights, change in CMJ/weighted CMJ, sprint time, heart rate and RPE were analyzed using a paired-sample t-test. The significance level was set at α < 0.05.

## 3. Results

### 3.1. Countermovement Jump Height

**Figure 2:**
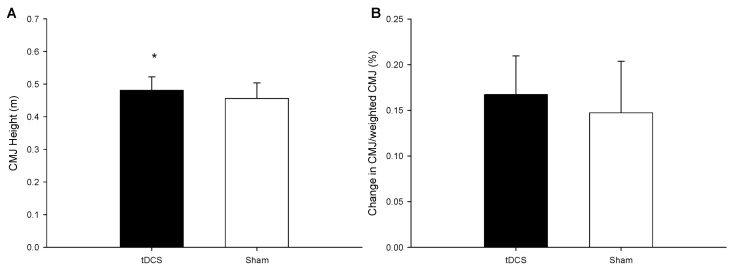
Countermovement jump height, and (**A**) the change in CMJ/weighted CMJ (**B**). * mean a significantly different between tDCS group and Sham groups. Values are mean ± SD.

The countermovement jump height of the participants is illustrated in Figure 2A. The countermovement jump height of the tDCS group was significantly higher than that of the sham group (*p* = 0.04). The weighted countermovement jump heights of the two groups were found to be non-significantly different, as shown in Figure 2B (*p* = 0.427).

### 3.2. Interval Sprint Exercise Performance

**Figure 3:**
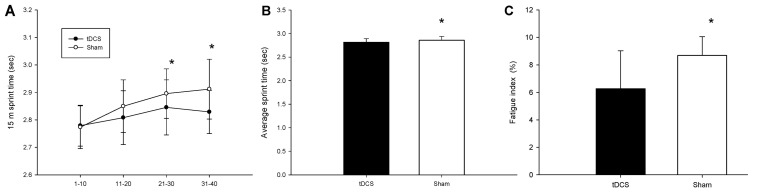
The completed time of sprinting on 1–10, 11–20, 21–30, and 31–40 m (**A**) Average time of sprinting (**B**) and fatigue index (**C**). * mean a significantly different between tDCS group and Sham group. Values are mean ± SD.

Figure 3A shows the average time taken to complete sprints 1–10, 11–20, 21–30, and 31–40. A significant interaction was found for the tDCS and sham trials (trial × time, *p* = 0.027). The time taken by the tDCS group to complete sprints 20–30 (*p* = 0.018) and 30–40 (*p* = 0.014) was significantly shorter than taken by the sham group. The tDCS group also took significantly less time on average overall (Figure 3B; *p* = 0.016). Figure 3C reveals that the fatigue index of the tDCS group was significantly lower than that of the sham group (*p* = 0.009).

### 3.3. Heart Rate during Sprinting and RPE Score

Table 1 shows the heart rates and RPE scores immediately after completion of sprints 1–10, 11–20, 21–30, and 31–40. The results showed that the heart rates (trial × time, *p* = 0.375) and RPE scores (trial × time, *p* = 0.642) of the two groups were not significantly different.

## 4. Discussion

This study clearly indicated that tDCS at 2 mA for 20 min on the motor cortex significantly improves the countermovement jump height and performance of the 40 15-m sprints of basketball players without influencing their heart rate during exercise or RPE score. In particular, tDCS significantly improved the sprint speed decline after 20 sprints that was caused by fatigue and helped participants maintain high sprint performance. In addition, the fatigue index during repeated sprinting was decreased.

This study is the first to find that tDCS can reduce the magnitude of the speed decline during repeated sprinting, maintain high sprint performance, and decrease fatigue. Early research found that tDCS can help people last longer in single-joint isometric exercise [10,11]. Contrary to single-joint exercise, sprinting is full-body multijoint exercise. High sprinting speed and phosphagen system capacity is crucial for basketball players. Researchers have previously only found that tDCS improves the power of the lower limbs [6] and cycling sprinting performance [14]. Similar to this study, Huang had participants perform a 6-s sprint for five rounds on a cycle ergometer with a 24-s break between rounds; the results revealed that tDCS did not influence performance in the first round but resulted in the mean power output in rounds 2–5 being significantly maintained [8]. In the present study, the jump height of countermovement jump was significantly higher in tDCS group than in the sham group, meaning that the power output in tDCS group improved as the countermovement jump has been suggested to predict the power of the lower limbs [17]. In addition, the 40 × 15-m sprints were used to determine the influence of tDCS on the sprinting performance of trained basketball players. This test method has previously been found to be related to the phosphagen system capacity of players of sports with interval-like characteristics [13]. The present results demonstrated that tDCS helped participants maintain high sprint speed during sprints 21–40. It may be related to tDCS increased the jump height of countermovement jump, exciting the motor cortex of the brain, increasing corticospinal output, and increasing the excitability of the motor cortex area [18]. Despite the exact mechanism behind the improvement remains unclear, however, the results of this study suggested that sprint performance has been shown to improve by tDCS especially after 20 sprints and helped the participants maintain high sprint performance.

Another possible reason for the apparent reduction in sprint speed decline and decrease in fatigue index may be related to the central nervous system and neuromuscular fatigue. In one study, participants were tested by performing eight cycling sprints and then submaximal exercise until exhaustion; tDCS was discovered to, at the same level of power output, inhibit central nervous system fatigue and improve endurance exercise performance [19]. In a study similar to the present research, tDCS led to participants maintaining their cognitive functions after sprinting and reporting less fatigue in the central nervous system [8]. In the present study, the countermovement jump height before sprinting of the tDCS group was higher than that of the sham group. Performance in the countermovement jump has been verified to be related to neuromuscular fatigue [20]. This study cannot provide direct evidence that explains the higher sprinting performance of the tDCS group. Nonetheless, this study provides new research evidence and shows that tDCS can help athletes maintain a relatively high level of sprinting performance.

The results in this study revealed that the heart rate and RPE score during sprinting were unaffected by tDCS during the sprinting. This is consistent with the results of past research. Studies involving different types of exercise intervention, including swimming, running, and cycling have found that tDCS does not influence the heart rate during exercise [14,21,22]. Additionally, a double-blind, randomized, and counterbalanced study discovered that tDCS did not influence the postexercise perceived fatigue of trained long-distance runners [23]. Park et al. found that tDCS improved performance in endurance exercise without influencing heart rate, RPE, or the metabolism index [14]. In addition, tDCS have been suggested to increase the recruitment of motor units [24] or increase the firing rate [25] measured by EMG. These reasons may provide the other explanations for no influencing heart rate and RPE in this study. In the future, further research might explore in depth the correlations between tDCS, central nervous system fatigue, and exercise performance.

The major limitation of this study is that the activation states of the M1 area were not confirmed using functional magnetic resonance imaging after tDCS was conducted. Consequently, tDCS may have activated cortex areas other than M1. In one study, tDCS was verified using functional magnetic resonance imaging to activate the M1 area [1]. Consequently, the authors of this study believe that the tDCS, conducted in this study, activated the M1 area, and improved sprint performance.

## 5. Conclusions

This study clearly verified that tDCS alleviates the speed decline during sprinting, decreases fatigue, and increases countermovement jump height without influencing heart rate or RPE. These results implicated that tDCS significantly improved the sprint speed decline after 20 sprints that was caused by fatigue and helped the participants maintain high sprint performance. The athletes and coaches of sports with interval-like characteristics a method of improving exercise performance in training and competition in the future.

## Figures and Tables

**Table 1:** Heart rate during sprinting and RPE score. Values are mean ± SD, *n* = 13. tDCS, transcranial direct currentstimulation group; Sham, Sham group.

	Pre-Sprint	1–10	11–20	21–30	31–40
Heart Rate					
tDCS	105.8 ± 25.8	159.5 ± 18.0	167.5 ± 13.6	175.1 ± 7.8	171.5 ± 17.4
Sham	101.7 ± 20.4	167.1 ± 10.0	172.9 ± 5.6	174.8 ± 7.9	173.9 ± 9.2
RPE					
tDCS	1.0 ± 0.0	3.63 ± 1.0	5.64 ± 0.8	7.54 ± 0.9	8.91 ± 0.8
Sham	1.0 ± 0.0	3.73 ± 1.4	5.91 ± 1.2	7.72 ± 0.9	8.72 ± 0.9

RPE—rating of perceived exertion, SD—standard deviation.

## Data Availability

Not applicable.
